# Effects of a text messaging smoking cessation intervention amongst online help-seekers and primary health care visitors: findings from a randomised controlled trial

**DOI:** 10.1186/s12916-023-03073-5

**Published:** 2023-10-04

**Authors:** Jenny Blomqvist, Katarina Ulfsdotter Gunnarsson, Preben Bendtsen, Marcus Bendtsen

**Affiliations:** 1 Department of Health, Medicine and Caring Sciences, Linköping University, 581 83 Linköping, Sweden; 2 Department of Medical Specialist, Motala, Sweden

**Keywords:** Smoking cessation, Telemedicine, General population, Randomised controlled trial

## Abstract

**Background:**

Smoking continues to be a leading risk factor for several diseases globally. We hypothesised that an intervention delivered via text messages could help individuals who were looking to quit.

**Methods:**

A two-arm, parallel-groups, randomised controlled trial was employed. Both groups received treatment as usual, with the intervention group also receiving a 12-week text messaging intervention. Participants were adult, weekly or more frequent smokers, recruited online and in primary health care centres. Research personnel were blinded, while participants were not. Primary outcomes were prolonged abstinence and point prevalence of abstinence, 3 and 6 months post-randomisation. All randomised participants were included in analyses.

**Results:**

Between 18 September 2020 and 16 June 2022, we randomised 1012 participants (intervention: 505, control: 507). Outcome data was available for 67% (*n* = 682) of participants at 3 months and 64% (*n* = 643) at 6 months. At 3 months, the odds ratio (OR) of prolonged abstinence was 2.15 (95% compatibility interval [CoI] = 1.51; 3.06, probability of effect [POE] > 99.9%, *p* < 0.0001), and for point prevalence of abstinence, it was 1.70 (95% CoI = 1.18; 2.44, POE = 99.8%, *p* = 0.0034) in favour of the text messaging intervention. At 6 months, the OR of prolonged abstinence was 2.38 (95% CoI = 1.62; 3.57, POE > 99.9%, *p* =  < 0.0001), and for point prevalence, it was 1.49 (95% CoI = 1.03; 2.14, POE = 98.3%, *p* = 0.0349) in favour of the text messaging intervention. Analyses with imputed data were not markedly different.

**Conclusions:**

Amongst general population help-seekers—who on average had smoked for 25 years—access to a 12-week text messaging intervention produced higher rates of self-reported smoking abstinence in comparison to treatment as usual only. The intervention could be part of the societal response to the burden which smoking causes; however, findings are limited by risk of bias due to attrition, self-reported outcomes, and lack of blinding.

**Trial registration:**

The trial was preregistered in the ISRCTN registry on 27/07/2020 (ISRCTN13455271).

**Supplementary Information:**

The online version contains supplementary material available at 10.1186/s12916-023-03073-5.

## Background

There are 1.14 billion tobacco smokers globally, with 7.41 trillion cigarette-equivalents being consumed annually [1]. The number of cigarettes smoked per day is a risk factor of many diseases, for example being ranked the leading risk factor of cancer globally [2]. The impact of smoking is severe, with 7.69 million deaths (13.6% of all deaths in 2019) being attributable to smoking [1], and tobacco is globally ranked the most severe risk factor for disability adjusted life years for men, and the seventh for women [1, 3].

Data from the Public Health Agency in Sweden showed that in 2021 [4], approximately 6.1% of the population aged between 16 and 84 were daily consumers of tobacco cigarettes amongst both men and women. Additionally, 4.6% of the age group report consuming tobacco cigarettes occasionally. In 2003, the percentage of daily smokers was 24% amongst women and 19% amongst men, indicating a steady decrease over the last two decades [5]. Though decreasing in prevalence over the last decades, smoking remains one of the leading risk factors for multiple diseases, including chronic obstructive pulmonary disease, stroke, pulmonary cancer, and myocardial infarction—all amongst the leading causes of deaths in Sweden and globally [6]. We are closer than ever to eradicating one of the leading causes of disease in Sweden; however, since smoking still is prevalent, and young individuals still start smoking [7], there is a need for effective smoking cessation interventions that can scale to a national level and are designed to reach individuals requiring smoking cessation support in the general population.

Digital interventions may contribute to the societal response to reduce the prevalence of smoking. These interventions are characterised by delivering cessation support materials using text messages, e-mail, mobile phone apps, etc. Text messaging-based interventions are particularly important since they rely on standard technology, which is increasingly prevalent globally, and can be delivered at relatively low cost. This means that they may be able to reach further into the community than face-to-face interventions. Early trials of text messaging-based interventions showed promising results, notably the txt2stop trial (*n*= 5800) [8], which found increased abstinence amongst those with access to the intervention: biochemically verified abstinence odds ratio [OR] = 2.20, 95% confidence interval [CI] = 1.80; 2.68, *p* < 0.001 and self-reported abstinence OR = 1.47, 95% CI 1.40; 1.66, *p*< 0.001). Since then, trials have been conducted internationally of text messaging interventions, targeting different populations, and a body of evidence has been produced which support their use [9, 10].

In Sweden, there have been two trials of text messaging interventions. Both have targeted younger individuals in well-defined settings: the first amongst college and university students [11] and the second amongst high school students [12, 13]. The trials found strong evidence of the effectiveness of the interventions, which is encouraging as prevention at an early age is important. However, there have been no studies in Sweden which target the general population. It is uncertain if this type of intervention also can help older individuals, who have smoked for longer, and may already have tried quitting multiple times using existing support. Therefore, this study aimed to investigate if a text messaging intervention could help individuals amongst the general population who were looking to quit smoking.

## Methods

We conducted a 2-arm RCT with parallel-groups (1:1), following a Bayesian sequential design. The trial was preregistered in the ISRCTN registry on 27/07/2020 (ISRCTN13455271) and received ethical approval from the Swedish Ethical Review Authority on 16/06/2020 (Dnr 2020–01427). A trial protocol was submitted prior to enrolment [14], and there were no deviations from the protocol. This report follows the recommendations of the CONSORT statement [15].

### Participants

Recruitment took place in two different settings. First, online advertisement (Google, Bing, and Facebook) was used to recruit individuals seeking help to quit smoking. Individuals clicking on the advert were taken to the study website which contained study information and instructions on how to sign up. Second, health care professionals at 54 participating primary health care units in the south of Sweden advertised the trial to patients through printed media (e.g. flyers, leaflets, business cards, posters). Like the website, the printed media contained study information and instructions on how to sign up. Regardless of setting, individuals were instructed sign up for the trial by sending a text message to a dedicated telephone number. Within 5 min, they received a text message in response, with a hyperlink to study information and an informed consent form.

Participants consenting to take part in the trial were immediately redirected to a baseline questionnaire, after which eligible participants were randomised. Individuals aged 18 years or older who smoked at least 1 cigarette per week were eligible for the trial. Most of the study information, and all questionnaires, were delivered to participants through their mobile phone and was in Swedish; thus, participants without access to a mobile phone and who did not comprehend Swedish well enough to sign up for the trial were excluded.

### Interventions

Both intervention and control groups were given treatment as usual, and neither were restricted from using other available smoking cessation aids. The intervention group were in addition given access to a text messaging intervention. Treatment as usual was in this trial defined as follows. For participants recruited through online advertisements, it was defined as referral to a nationally available smoking cessation helpline and a nationally organised website with general information about smoking and health. For participants recruited through primary health care units, it was defined as the same type of referral as participants recruited online, with additional referral from the primary health care units to have a conversation with a nurse or smoking cessation specialist about smoking cessation and health.

Two versions of the intervention were available: one general version and one that was tailored specifically for individuals undergoing elective surgery. Both versions were based on findings from our previous research [11–13]. The elective surgery intervention was allocated to participants in the intervention group who reported having elective surgery planned in the next 3 months. Both versions of the intervention consisted of a 12-week text message program with messages sent to participants’ mobile phones daily.

Over the first few weeks, participants received 2–4 messages per day, which was reduced to 2 messages per day during the middle part of the intervention, and further reduced to 1 message per day during the latter part of the intervention. The content of the messages was primarily informational and encouraging, and some messages asked participants to do specific tasks, such as throw away ashtrays. None of the messages asked participants to respond, but participants could request extra supportive messages by sending a text message with one of three keywords: weight, relapse, or craving. A message was then sent back to participants with specific information about potential weight gain, what to do if one relapses, or help if they were experiencing nicotine cravings. Unique for the elective surgery version was that additional messages about complications and recovery from surgery were included, and some of the messages included hyperlinks to web-based modules which contained materials specifically designed for the surgery context. Please see the study protocol for more details [14].

### Baseline measures

At baseline, participants were asked to complete an electronic questionnaire on their mobile phones. The questionnaire asked about the following: gender, age, the number of years smoking, monthly/weekly/daily smokers and the number of cigarettes typically smoked in this interval, lifetime number of quit attempts, cessation counselling experience, use of quit smoking helpline, and use of snus (which is a moist oral tobacco product which is common in Sweden, sometimes translated as *snuff*). In addition, participants completed the Fagerström Test for Nicotine Dependence scale (FTND) [16], which scores participants from 1 to 10, where higher scores indicate higher nicotine dependence. Due to snus being commonly used in Sweden, the scale was amended to include one item asking about the use of snus. The item asked: *Do you use snus?*, with response options: *No*; *A few times a month*; *A few times a week*; *Daily – less than 1/3 of a box*; *Daily – 1/3 of a box*; *Daily – 1/2 of a box*; and *Daily – 1 box or more*. For scoring purposes, a full box per day or more scored 3 points, half a box daily scored 2 points, third of a box daily scored 1 point, and the other response options scored 0 points. The total FTND score was calculated using either the snus item or the standard cigarette item, whichever the participant scored higher on. Note that only 21 participants (2.1%) scored higher on the snus item. The internal reliability of the FTND for this sample was assessed using Cronbach’s alpha, which resulted in an alpha of 0.67. This is below what is typically considered a fair reliability for clinical purposes, although note that here we only use it to adjust our primary analyses for baseline dependence and not as an outcome measure. The FTND scale has not been validated in the context of this study; however, scores ranging between 5 and 7 have previously been used to indicate moderate nicotine dependence and 8 + to indicate high nicotine dependence.

### Outcomes

The primary outcomes were prolonged abstinence and point prevalence of smoking abstinence, measured at 3 and 6 months post-randomisation. Prolonged abstinence was defined following the Russell standard [17] definition of not having smoked more than 5 cigarettes in the past 8 weeks (thus allowing for a 4-week grace period). The abstinence period was adjusted to 5 months at the 6-month follow-up. Point prevalence of smoking abstinence was defined as not smoking any cigarette during the past 4 weeks, as recommended by the Society for Research on Nicotine and Tobacco [18].

Secondary outcomes were 7-day point prevalence of smoking abstinence, number of cigarettes smoked weekly (if still smoking), number of quit attempts since baseline, and number of uses of other smoking-cessation aids since baseline.

Three potential mediating factors were assessed: importance, confidence, and knowledge of how to quit (know-how). To reduce participant burden across the entire trial period, we decided against using multi-item questionnaires to measure these factors, instead relying on face valid single-item measures based on importance and confidence rulers [19]. The items used are presented in Table 1. Findings from mediator analyses based on these outcomes will be reported separately; however, due to limited evidence of construct validity, caution is advised in interpreting findings based on these mediators.

**Table 1:** Items used to assess three potential mediators

• **Importance:** How important is it for you to quit smoking?
• (10-point scale ranging from 1 = “Not important” to 10 = “Very important”)
• **Confidence**: How confident are you that you will be able to quit smoking?
• (10-point scale ranging from 1 = “Not at all” to 10 = “Very confident”)
• **Know-how:** How well do you know how to quit smoking?
• (10-point scale ranging from 1 = “Not well at all” to 10 = “Very well”)

There were three follow-up intervals: 1, 3, and 6 months post-randomisation. At the 1-month follow-up, the three hypothesised mediators were assessed only. All follow-ups were initiated by sending text messages to participants with hyperlinks to questionnaires. In all cases, the following additional attempts were made to collect data:

1. A total of 2 reminders were sent 2 days apart.
2. If no response was given to (1), then questions were sent directly in a text message, asking participants to respond directly with a text (no hyperlink). We only asked for primary outcome measures at this stage.
3. If there was no response given to (2) at 3 and 6 months, we attempted to call participants to collect primary outcomes. A maximum of 5 call attempts were made.

### Randomisation and blinding

Allocation was done according to a computer-generated random sequence. Participants were stratified according to which of the two versions of the intervention was appropriate (general or surgery). Block randomisation was used to ensure equal number of participants in each group within stratum, using random block sizes of 2 and 4. Randomisation was done immediately after participants responded to the baseline questionnaire using their mobile phone. Once a response was received by the backend server, allocation took place automatically, and participants were told about group allocation via a text message. Research personnel were not able to affect the allocation and all study procedures were automated, preventing subversion of allocation concealment.

Participants were aware of their group allocation; however, research personnel were blinded. All questionnaires were completed by participants on their own mobile phones, without supervision by research personnel. Non-responders were called, and during the call, it was possible that participants revealed their allocation to assessors. This means that there was a risk of detection bias (see Limitations).

### Statistical analysis

The statistical methods applied were pre-specified in the trial protocol [14]. All participants were included in the analyses in the groups to which they were randomised (intention-to-treat). Missing data was initially handled by available case analysis under the missing at random (MAR) assumption. Outcome data missing systematically due to the outcome itself will invalidate the MAR assumption; thus, evidence of such was sought in attrition analyses. Sensitivity analyses that include imputed values for missing outcome data were also conducted.

All models were estimated using Bayesian inference [20]. We use the median of the posterior distribution as a point estimate of effect and report 95% compatibility intervals (CoI) defined by the 2.5% and 97.5% percentiles of the posterior distribution. We complemented the Bayesian analyses with null hypothesis tests at the 0.05 significance level.

#### Primary and secondary outcomes

For the primary and secondary outcome measures, differences between the two groups at the follow-up intervals with respect to prolonged abstinence and 4-week and 7-day point prevalence of abstinence were analysed using logistic regression. Negative binomial regression was used to analyse the number of quit attempts, use of other smoking cessation services, and cigarettes smoked weekly (amongst those who still smoked). Models were adjusted for baseline characteristics (gender, age, nicotine dependence, importance, confidence, and know-how) as well as the stratifying variable in the randomisation procedure (general or surgery eligibility).

Effect modification analyses were performed for the primary outcomes, exploring interactions between group allocation and baseline variables. We also estimated effect modification based on which setting participants were recruited from (online or primary health care) and which version of the intervention they were eligible for (general or surgery).

#### Attrition analyses

Analyses comparing responders and non-responders with respect to baseline variables were conducted using logistic regression. We used Cauchy priors for covariates, with a standard normal hyperprior for the scale parameter to induce shrinkage. Models were estimated with and without interaction terms between baseline variables and group.

Based on the assumptions of repeated attempt models [21, 22], a second attrition analysis investigated if late responders were different than early responders with respect to primary outcomes. An association between attempts to collect follow-up and outcomes could in such a case imply systematic differences between non-responders and responders. We regressed primary outcomes against the number of attempts to collect follow-up data before a response was recorded and an interaction term with group.

#### Sample size

We used a Bayesian sequential design to monitor recruitment [23]. As data became available, the primary outcomes were modelled according to the analysis plan and the coefficient for group allocation was assessed for effect, harm, and futility. Letting *ß*_*k*,*i*_ represent the regression coefficient for group allocation at time *k* for outcome *I*, and *D* represent the accumulated data, the target criteria were as follows:

- Effect: *p*(*ß*_*k*,*i*_ > 0 | *D*) > 97.5% and *p*(*ß*_*k*,*i*_ > log(1.3) | *D*) > 50%
- Harm: *p*(*ß*_*k*,*i*_ < 0 | *D*) > 97.5% and *p*(*ß*_*k*,*i*_ < log(1/1.3) | *D*) > 50%
- Futility: *p*(log(1/1.3) < *ß*_*k*,*i*_ < log(1.3) | *D*) > 95% for futility.

For the effect and harm criteria, we used a standard normal prior for covariates (mean = 0, SD = 1) and a slightly wider prior was used for the futility criterion (mean = 0, SD = 2). The criteria are targets; thus, at each interim analysis, we evaluated each target for each covariate and decided to continue or stop recruitment. Note that this Bayesian approach allows us to look at the data an unlimited number of times without worrying about multiplicities and error rates, as would be necessary using a frequentist approach. Also, since no fixed sample size is prespecified, we reduced the risk of stopping both too early and too late [23, 24].

## Results

**Fig. 1:**
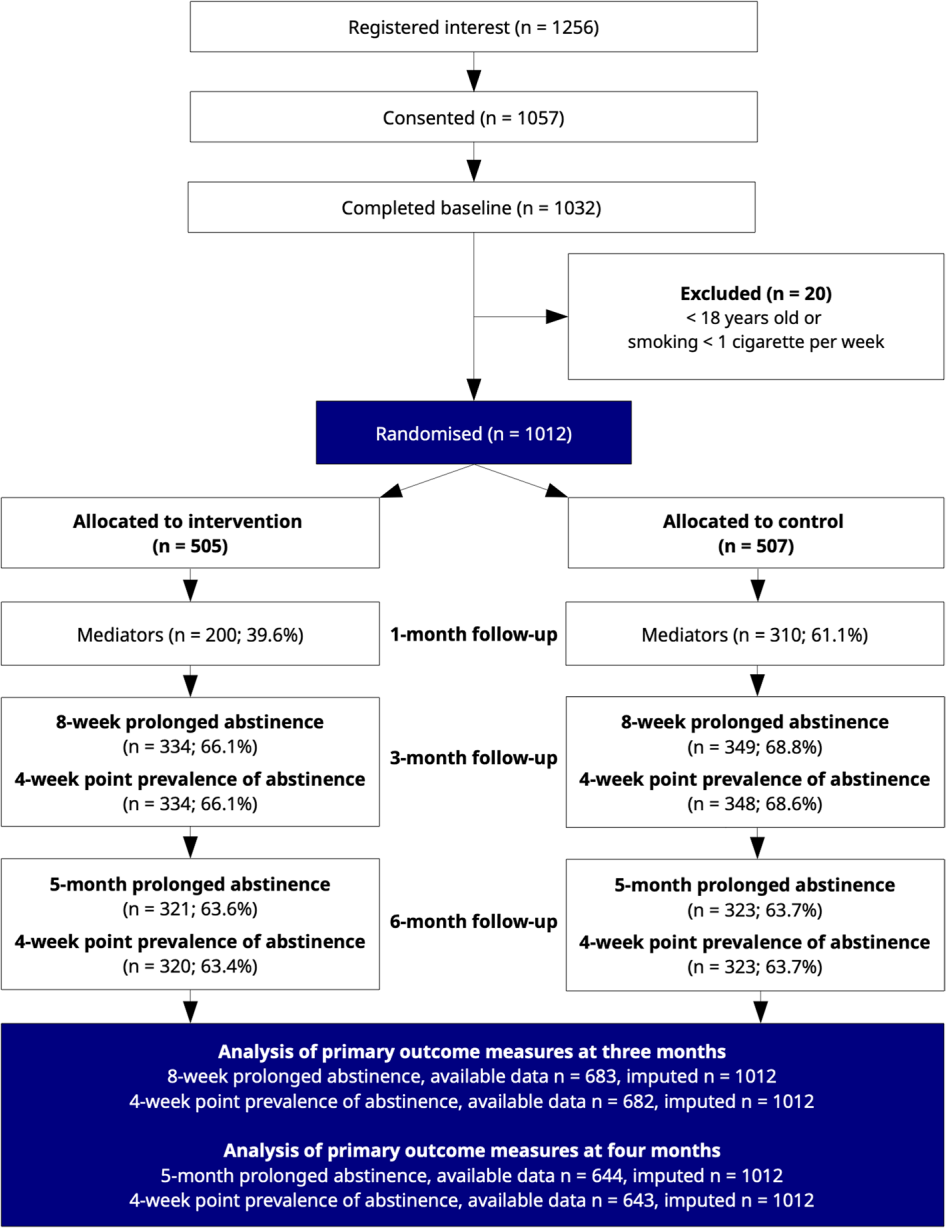
CONSORT flow diagram showing numbers signed up, consented, randomised, followed-up, and analysed

Between 18 September 2020 and 16 June 2022, we randomised 1012 participants (intervention: 505, control: 507). At this time, the target criteria for recruitment were sufficiently achieved and recruitment stopped (see Additional File 1). A total of 1256 individuals registered interest in the trial, of which 199 did not consent, 25 did not complete baseline, and 20 did not fulfil the inclusion criteria. Almost all participants, 95.9% (971/1012), were recruited through online advertisements, and 6.2% of participants (63/1012) reported at baseline having surgery planned within 3 months. Amongst intervention group participants, 23% (116/505) stopped the messages before the 12-week program was complete, on average 25 days after starting. All participants were contacted at follow-up and included in the analyses in the groups to which they were randomised. In Table 2, baseline characteristics of participants are presented, and a CONSORT flow diagram can be found in Fig. 1.

**Table 2:** Baseline characteristics of randomised participants

	**Total (*n* = 1012)**	**Intervention (*n* = 505)**	**Control (*n* = 507)**
Woman^a^, *n* (%)	820 (81)	406 (80.4)	414 (81.7)
Age, mean (SD)	45.4 (14)	45 (13.9)	45.7 (14.1)
Years of smoking, mean (SD)	25.3 (14.6)	24.7 (14.3)	25.9 (14.9)
Daily smokers (vs. weekly smokers), *n* (%)	981 (96.9)	489 (96.8)	492 (97.0)
Cigarettes smoked per week, mean (SD)	101 (46.2)	101.4 (47.3)	100.6 (45.1)
**Use of snus**^**b**^**, *n* (%)**			
Daily	63 (6.2)	27 (5.3)	36 (7.1)
Weekly or monthly	90 (8.9)	45 (8.9)	45 (8.9)
No	859 (84.9)	433 (85.7)	426 (84.0)
Fagerström test for nicotine dependence, mean (SD)	5 (2.2)	5 (2.2)	5 (2.2)
Quit attempts^c^, mean (SD)	7.2 (13.7)	7.0 (12.7)	7.5 (14.6)
**Cessation counselling experience, *n* (%)**			
Yes, now	32 (3.2)	13 (2.6)	19 (3.7)
Yes	192 (19.0)	95 (18.8)	97 (19.1)
No	788 (77.9)	397 (78.6)	391 (77.1)
Used quit smoking helpline, *n* (%)	13.6 (138)	14.5 (73)	12.8 (65)
Importance of quitting^d^, mean (SD)	9.4 (1.3)	9.4 (1.3)	9.5 (1.2)
Confidence in ability to quit^d^, mean (SD)	6.2 (2.5)	6.3 (2.5)	6.2 (2.6)
Knowledge of how to quit^d^, mean (SD)	5.5 (2.6)	5.5 (2.7)	5.5 (2.5)

^a^The baseline questionnaire included a category “Other”; however, it was not chosen by any participant

^b^Snus is a moist oral tobacco product which is common in Sweden, sometimes translated as *snuff*

^c^Participants were asked about the lifetime number of quit attempts

^d^Three single item measures were used to assess importance, confidence, and know-how regarding smoking cessation. Responses ranged from 0 to 10, with 10 representing highest agreement (i.e. very important, very confident, very knowledgeable). The same items were used at follow-up as hypothesised mediators of effects

### Primary and secondary outcomes

**Fig. 2:**
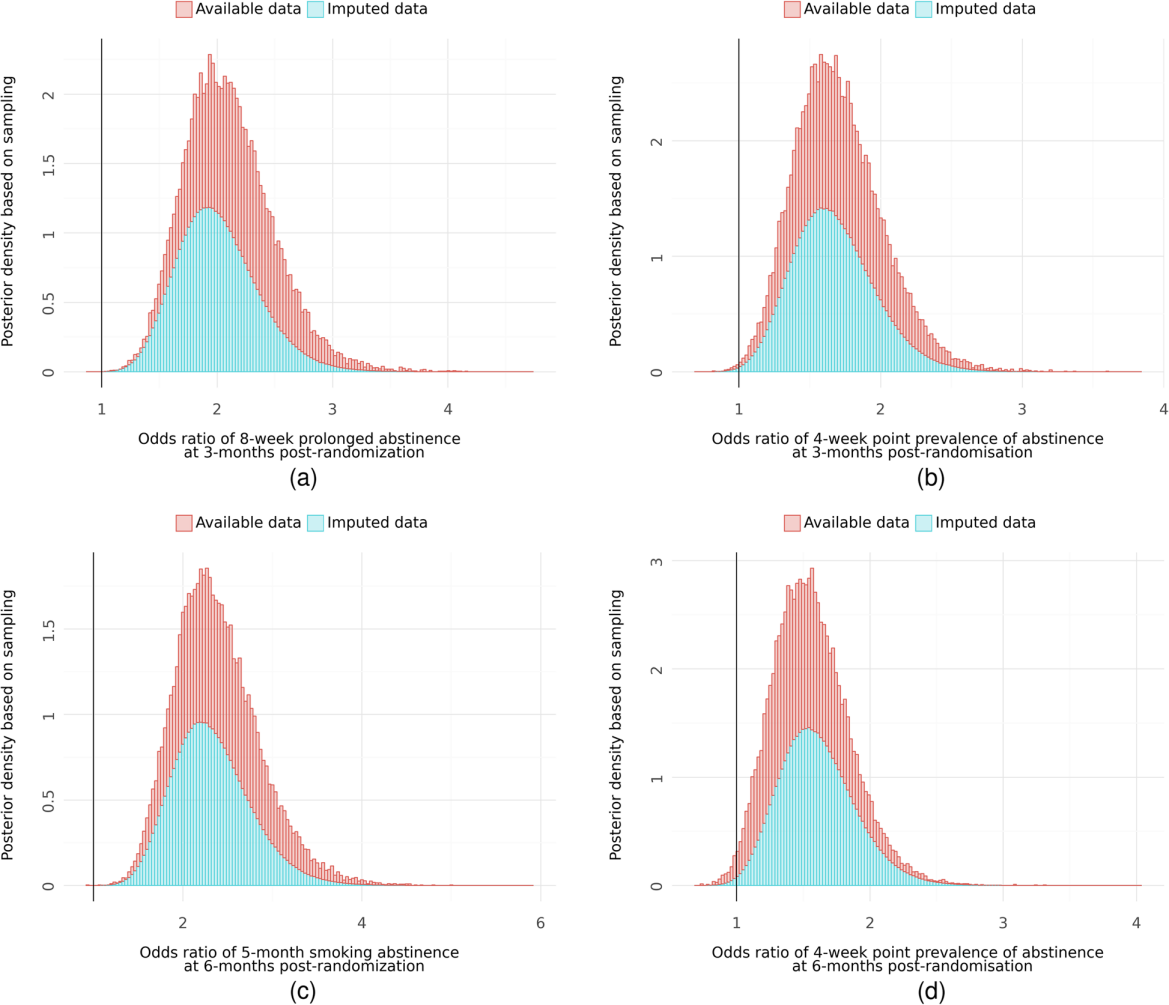
Posterior density of effect estimates (odds ratios) comparing intervention to control: (**a**) 8-week prolonged abstinence at 3 months, (**b**) 4-week point prevalence of abstinence at 3 months, (**c**) 5-month prolonged abstinence at 6 months, and (**d**) 4-week point prevalence of abstinence at 6 months

Descriptive statistics of primary and secondary outcomes are presented in Table 3. Posterior densities of primary outcome effect estimates are depicted in Fig. 2, and point estimates are presented in Table 4. Point estimates of effects for secondary outcomes are presented in Table 5. Overall, smoking abstinence was markedly higher in the intervention group, both at 3 and 6 months post-randomisation. This is evident when looking at the posterior densities of odds ratios in Fig. 2, as they are shifted to the right of one, and the posterior probability of effect is high in all cases in Table 4. Amongst those still smoking, the number of cigarettes smoked per week were lower in the intervention group at both follow-up intervals, and the number of quit attempts were higher in the intervention group at 3 months. There was no observed difference in use of other support between groups.

**Table 3:** Descriptive statistics of primary and secondary outcomes

	**Intervention**^**a**^	**Control**^**a**^
**Three months post-randomisation**		
8-week prolonged abstinence	32.9% (110/334)	18.9% (66/349)
4-week point prevalence of smoking abstinence	28.4% (95/334)	19.0% (66/348)
7-day point prevalence of smoking abstinence	47.2% (116/246)	30.2% (83/275)
Cigarettes smoked weekly (amongst smokers only)	60.9 (47.8) *n* = 117	82.5 (49.1) *n* = 188^b^
Quit attempts since baseline	5.5 (14.4) *n* = 213	2.3 (4.7) *n* = 250
Use of additional support since baseline	0.7 (0.6) *n* = 334	0.7 (0.5) *n* = 349
**Six months post-randomisation**		
5-month prolonged abstinence	29.3% (94/321)	14.6% (47/323)
4-week point prevalence of smoking abstinence	27.8% (89/320)	20.1% (65/323)
7-day point prevalence of smoking abstinence	50.9% (115/226)	28.1% (70/249)
Cigarettes smoked weekly (amongst smokers only)	68.6 (45.4) *n* = 90	84.5 (53.1) *n* = 164^b^
Quit attempts since baseline	3.6 (9.1) *n* = 186	3.3 (9.9) *n* = 221
Use of additional support since baseline	0.6 (0.6) *n* = 221	0.7 (0.6) *n* = 323

^a^For binary variables: proportion of events using available data (count of events/count of available data). For numeric data: mean (SD) using available data, and *n* = count of available data

^b^The notable difference in available data between groups for cigarettes smoked weekly is due to more participants in the control group continuing to smoke. Only smokers were asked this question

**Table 4:** Point estimates of effects on primary outcomes comparing intervention versus control

	**Available data analysis**			**Imputed data analysis**		
	**Estimate**^**a**^** (95% CoI)**	**Prob.**^**b**^ **OR > 1**	***p*-value**^**c**^	**Estimate**^**a**^** (95% CoI)**	**Prob.**^**b**^ **OR > 1**	***p*-value**^**c**^
**Three months post-randomisation (intervention versus control)**						
8-week prolonged abstinence	2.15 (1.51; 3.06)	> 99.9%	< 0.0001	1.94 (1.38; 2.75)	> 99.9%	< 0.0001
4-week point prevalence of abstinence	1.70 (1.18; 2.44)	99.8%	0.0034	1.68 (1.19; 2.37)	99.8%	0.0033
**Six months post-randomisation (intervention versus control)**						
5-month prolonged abstinence	2.38 (1.62; 3.57)	> 99.9%	< 0.0001	2.31 (1.59; 3.39)	> 99.9%	< 0.0001
4-week point prevalence of abstinence	1.49 (1.03; 2.14)	98.3%	0.0349	1.62 (1.14; 2.30)	99.6%	0.0069

^a^The median of the posterior distribution over odds ratios, with 2.5% and 97.5% percentiles representing a compatibility interval (CoI)

^b^The proportion of the posterior distribution over odds ratios which is greater than 1

^c^*P*-values are based on maximum likelihood estimation

**Table 5:** Point estimates of effects on secondary outcomes comparing intervention versus control

	**Available data analysis**			**Imputed data analysis**		
	**Estimate**^**a**^** (95% CI)**	**Prob.**^**b**^ **OR > 1**	***p*-value**^**c**^	**Estimate**^**a**^** (95% CI)**	**Prob.**^**b**^ **OR > 1**	***p*-value**^**c**^
**Three months post-randomisation (intervention versus control)**						
7-day point prevalence of abstinence	2.00 (1.41; 2.86)	> 99.9%	0.0002	1.75 (1.26; 2.43)	> 99.9%	0.0009
Cigarettes smoked weekly (amongst smokers only)	0.73 (0.62; 0.86)	> 99.9%	0.0002	0.73 (0.60; 0.87)	> 99.9%	0.0005
Quit attempts since baseline	2.20 (1.69; 2.90)	> 99.9%	< 0.0001	1.69 (1.22; 2.30)	99.9%	0.0012
Use of additional support since baseline	0.93 (0.77; 1.11)	79.8%	0.409	0.91 (0.77; 1.07)	87.6%	0.2416
**Six months post-randomisation (intervention versus control)**						
7-day point prevalence of abstinence	2.56 (1.73; 3.77)	> 99.9%	< 0.0001	2.23 (1.57; 3.17)	> 99.9%	< 0.0001
Cigarettes smoked weekly (amongst smokers only)	0.78 (0.66; 0.93)	99.7%	0.0032	0.84 (0.71; 0.99)	98.2%	0.0427
Quit attempts since baseline	1.14 (0.88; 1.49)	84.4%	0.291	1.04 (0.73; 1.45)	58.8%	0.8375
Use of additional support since baseline	0..88 (0.73; 1.07)	89.1%	0.195	0.90 (0.75; 1.06)	89.9%	0.1994

^a^The median of the posterior distribution over odds ratios, with 2.5% and 97.5% percentiles representing a compatibility interval (CoI)

^b^The proportion of the posterior distribution over odds ratios which is greater than 1

^c^*P*-values are based on maximum likelihood estimation

### Ancillary analyses

#### Interactions

Interaction models revealed some attenuation of effects with respect to baseline variables. Most consistently, there was evidence that age was a moderator for both primary outcomes at the 3-month interval and for prolonged abstinence at the 6-month interval. These analyses suggests that older participants were more likely to benefit from the intervention, as the interaction term had an OR of 1.03 (95% CoI = 1.00; 1.05, probability of interaction [POI] = 98.9%) for prolonged abstinence at 3 months, 1.02 (95% CoI = 1.00; 1.04, POI = 96.6%) for point prevalence at 3 months, and 1.02 (95% CoI = 1.00; 1.05, POI = 97.1%) for prolonged abstinence at 6 months.

There was also evidence that the intervention was more effective amongst those who scored higher on the confidence item at baseline (being more confident about being able to quit). However, this was less consistent with the moderation effect only marked in point prevalence at 3 months (OR = 1.17, 95% CoI = 1.02; 1.34; POI = 98.7%) and prolonged abstinence at 5 months (OR = 1.13, 95% CoI = 0.98; 1.30, POI = 94.7%). Finally, evidence that men were less likely to benefit from the intervention was found with respect to point prevalence at 3- and 6 months, with the evidence weaker for the latter. The OR was 0.45 (95% CoI = 0.20; 1.04, POI = 96.8%) at 3 months and 0.53 (95% CoI = 0.22; 1.24, POI = 92.9%) at 6 months.

We found no evidence of interactions between group allocation and recruitment method nor with access to the general or the surgery version of the intervention. However, these analyses were limited by small sample sizes.

#### Attrition

**Fig. 3:**
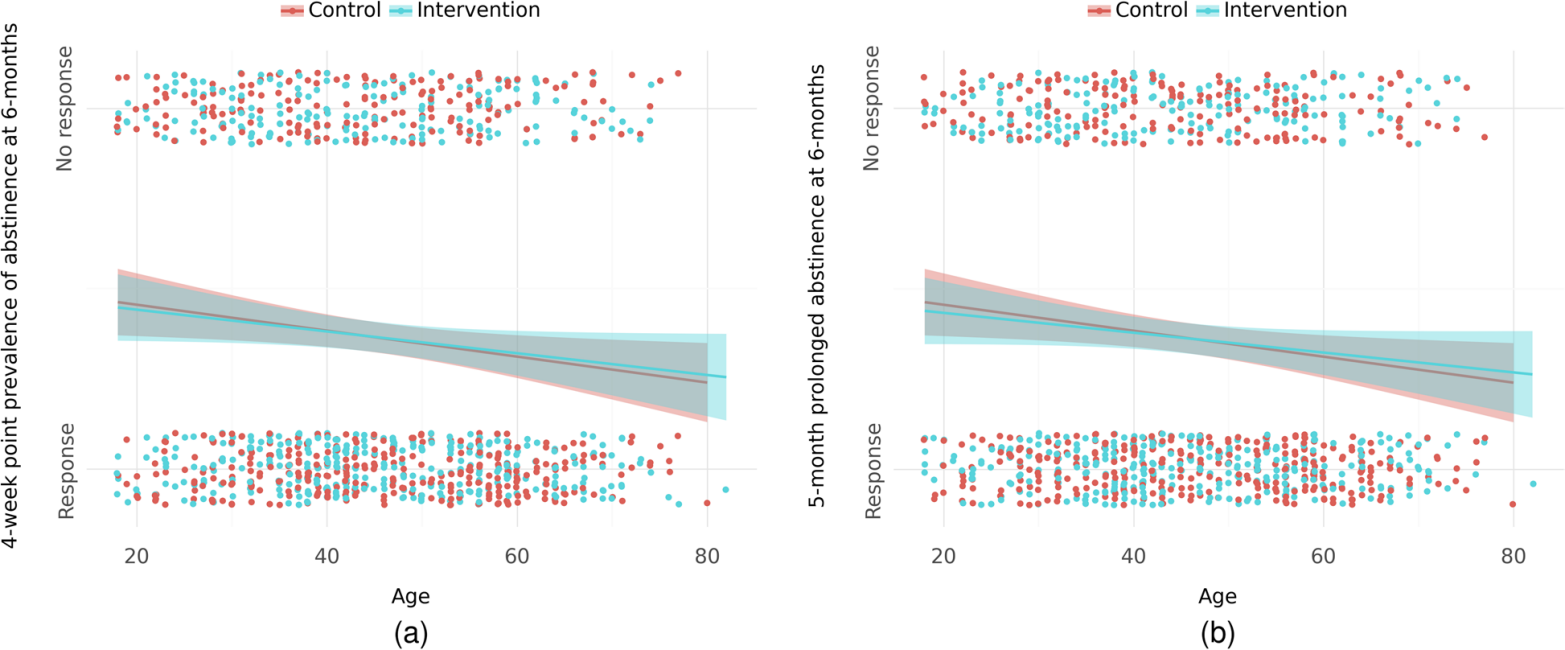
Response/No-response plotted against age at baseline and divided by group: (**a**) 5-month prolonged abstinence at 6 months and (**b**) 4-week point prevalence at 6 months

We found evidence that older participants in both groups were more likely to respond to follow-up at 6 months (se Fig. 3). For prolonged abstinence, the OR was 0.99 (95% CoI = 0.97; 1.00, probability of association [POA] = 97.6%), and for point prevalence, the OR was 0.99 (95% CoI = 0.97; 1.00, POA = 97.8%). No other marked associations between response and baseline characteristics, strata, or recruitment setting were found.

**Fig. 4:**
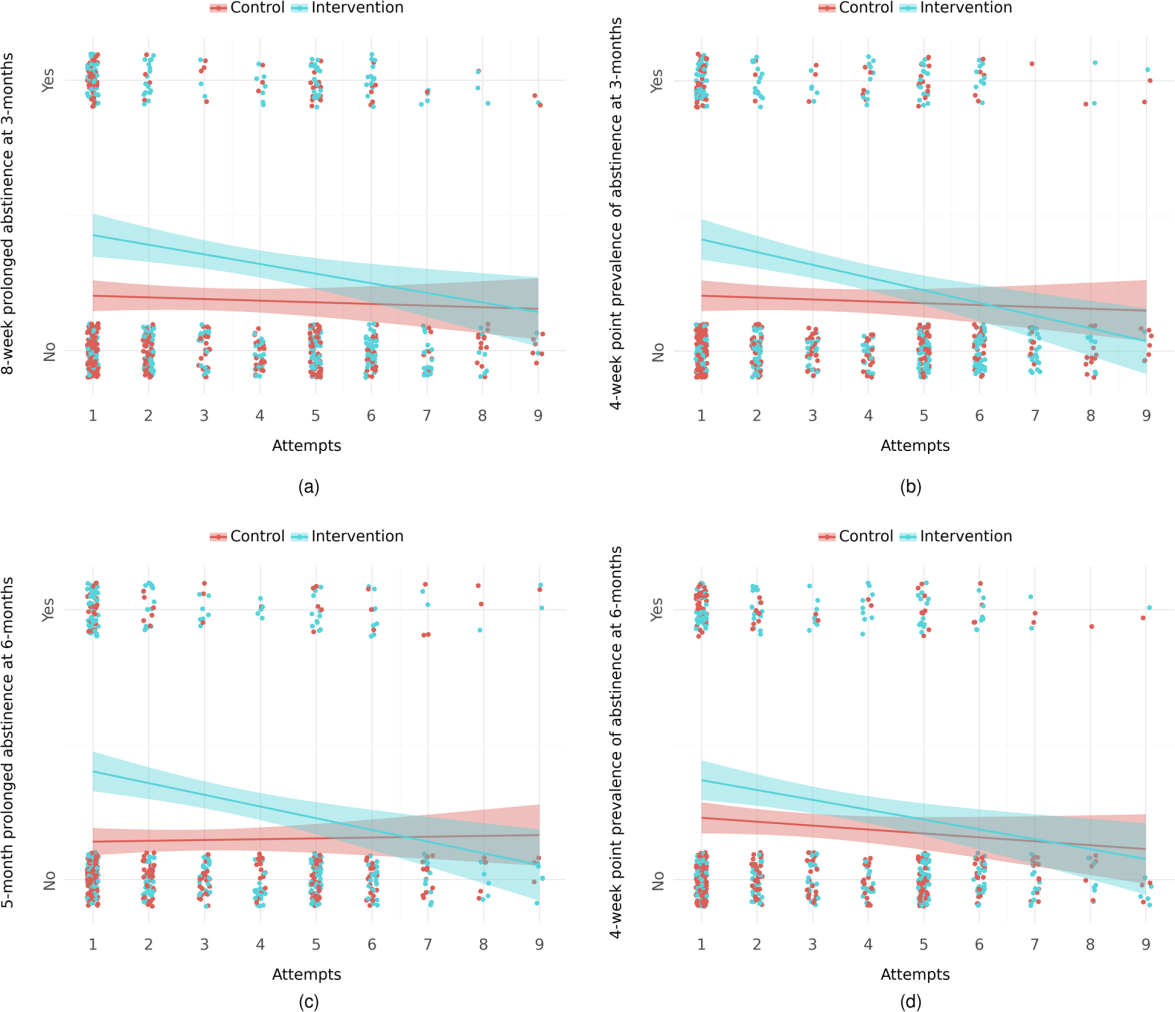
Primary outcomes plotted against number of attempts to collect data: (**a**) 8-week prolonged abstinence at 3 months, (**b**) 4-week point prevalence of abstinence at 3 months, (**c**) 5-month prolonged abstinence at 6 months, and (**d**) 4-week point prevalence of abstinence at 6 months

Associations between the number of attempts to collect data and primary outcomes are presented in Fig. 4. At 3 months, the association between attempts in the intervention group and prolonged abstinence had an OR of 0.85 (95% CI = 0.77;0.95, POA = 99.8%), and for point prevalence, the OR was 0.79 (95% CoI = 0.70; 0.88, POA > 99.90%). Similarly, at 6 months, the OR for attempts in the intervention group was 0.81 (95% CoI = 0.72; 0.91, POA > 99.9%) for prolonged abstinence and 0.83 (95% CoI = 0.73; 0.94, POA = 99.9%) for point prevalence of smoking abstinence. At 6 months, there was also evidence of an association between attempts and point prevalence in the control group, with an OR of 0.90 (95% CoI = 0.78; 1.02, POA = 95.1%).

## Discussion

We found evidence that access to a 12-week text messaging intervention produced higher self-reported smoking abstinence in comparison to treatment as usual only. Effects on abstinence were observed immediately after the intervention period and persisted 3 months later. In addition, amongst those who continued to smoke, those with access to the intervention reported a lower number of cigarettes smoked weekly. This is the first time the effectiveness of a text messaging smoking cessation intervention has been evaluated amongst the general population in Sweden, and while the point estimates of effect sizes were found to be larger than those of international studies, they are still comparatively similar. For instance, one meta-analysis of text messaging interventions reported an overall OR of 1.37 (95% CI = 1.25–1.51) for smoking abstinence [9]. Another meta-analysis [25], including digital smoking cessation intervention more broadly, found short-term ORs of 1.43 (95% CI = 1.09; 1.87) for prolonged abstinence and 1.75 (95% CI = 1.13;2.27) for 30-day point prevalence of abstinence, with long-term ORs for prolonged abstinence of 1.40 (95% CI = 1.19; 1.63). The estimated effects in this study are also comparable to those estimated in our own studies of text messaging smoking cessation interventions targeting high school and university students in Sweden [11–13].

Participants were long-time smokers (mean 25.3 years), with a history of multiple quit attempts (mean 7.2 attempts), yet the intervention was effective and showed no evidence of being less effective amongst those having smoked for longer. This is encouraging, as its important to both find ways of preventing individuals from becoming long time smokers and help those who have smoked for a long time to quit. All participants were referred to existing resources for smoking cessation, yet access to the intervention still improved abstinence rates. About a quarter of intervention group participants decided to stop the program prematurely. We did not require participants to give any reason for doing so (in accordance with ethical procedures); however, we may speculate that this was due to the program not suiting their needs, that they decided to abandon their quit attempt, or that they felt that they no longer needed support as they had successfully quit. On the other hand, more than three quarters of participants used the program to completion, seemingly finding it a helpful tool.

### Generalisability and limitations

Participants were recruited online and through primary health care centres, mimicking closely how the intervention could be disseminated in the real-world. Inclusion criteria were allowing, and few participants who showed interest in the trial were excluded. This pragmatic trial design leads us to interpret findings as estimates of effectiveness rather than efficacy, which strengthens the external validity of the trial. The design did however also limit our ability to blind participants, as they were naturally aware if they received the intervention or not. Although one could imagine some form of sham intervention with text messages containing non-smoking related information, it is unlikely that this would convince those seeking help that they had received cessation support.

Attrition rates were high in this trial, as is often the case with online trials with low thresholds of participation [26]. It was easy to join the trial even if one was simply curious about the trial itself with no intention of following through and likewise easy to forget that one was participating in a trial at all. We took measures to minimise attrition by using text reminders and calling for responses when necessary, however, even these strategies cannot guarantee full follow-up. Our sensitivity analyses using imputed data did not result in findings markedly different from those using available data. While age was associated with non-response, it was not consistent across all primary outcomes and follow-up intervals, and it was not differential between groups. There was however evidence that late responders in the intervention group were more likely to be self-reported smokers than late responders in the control group. While the assumption that late responders and non-responder are more alike than early responders cannot be tested, there is still a pattern of response which may suggest that the MAR assumption does not hold. Although speculative, it may be due to social desirability, where those who received the intervention felt that they were not living up to the researchers’ expectations of them, and thus did not want to respond to the questionnaires. If so, then estimates in our trial may be inflated. However, this analysis has its own limitations in terms of decreasing sample sizes as the number of attempts increase (se Fig. 4), and the model assumption of a monotonic relationship. Nonetheless, the estimates produced using imputed data, which uses available outcome data as auxiliary to predict missing outcome data, may therefore be interpreted as more conservative estimates of effectiveness.

Outcome measures were self-reported in this trial, which may be susceptible to recall bias and exacerbate the risk of social desirability bias. The Society for Research on Nicotine and Tobacco does however recommend that, in studies with limited face-to-face contact, it is neither required nor desirable to use biochemical verification [18], since it may introduce selection bias in who is tested. Lack of blinding of participants in combination with self-report does however introduce risk of performance bias, including both away and towards behaviour change due to disappointment about being in the control group [27, 28]. In addition, it is possible that those allocated to the intervention group were affected by social desirability to a greater degree than those in the control group, and therefore exaggerated their reports of non-smoking to conform to the aims of the trial, thereby biasing effect estimates away from the null.

All study procedures were automated, ensuring that the concealment of the allocation sequence could not be subverted nor that research personnel could become aware of allocation. However, to reduce attrition bias, we called participants who did not respond to our initial attempts to collect data. This induces a risk of detection bias, which has been shown to affect outcome reporting [29]. However, personnel who were making the calls were instructed to promptly ask the two primary outcome questions and collect responses and not probe participants about anything else. It is inevitable that some participants may have revealed their allocation anyway; however, we found that this risk of bias was minimal and worth taking as it decreased the risk of attrition bias.

## Conclusions

Access to a 12-week text messaging intervention produced higher self-reported smoking abstinence at 3 and 6 months post-randomisation amongst help-seekers in the general population. A pragmatic design allows for estimates to be interpreted as effectiveness, with risk of bias due to attrition, self-reported outcomes, and lack of blinding.

### Supplementary Information

**Additional file 1.** Recruitment criteria. **Table S1.** Evaluation of recruitment target criteria.

## Data Availability

A study protocol, including a statistical analysis plan, is available open-access [14]. Deidentified datasets generated during and/or analysed during the current study will be made available upon reasonable request to the corresponding author, after approval of a proposal and with a signed data access agreement.

## Abbreviations

CI: Confidence interval
CoI: Compatibility interval
MAR: Missing at random
OR: Odds ratio
POA: Probability of association
POE: Probability of effect
POI: Probability of interaction
RCT: Randomised controlled trial
SD: Standard deviation

## References

[1] Reitsma MB, Kendrick PJ, Ababneh E, Abbafati C, Abbasi-Kangevari M, Abdoli A (2021). Spatial, temporal, and demographic patterns in prevalence of smoking tobacco use and attributable disease burden in 204 countries and territories, 1990–2019: a systematic analysis from the Global Burden of Disease Study 2019. Lancet.

[2] Tran KB, Lang JJ, Compton K, Xu R, Acheson AR, Henrikson HJ (2022). The global burden of cancer attributable to risk factors, 2010–19: a systematic analysis for the Global Burden of Disease Study 2019. Lancet.

[3] Stanaway JD, Afshin A, Gakidou E, Lim SS, Abate D, Abate KH (2018). Global, regional, and national comparative risk assessment of 84 behavioural, environmental and occupational, and metabolic risks or clusters of risks for 195 countries and territories, 1990–2017: a systematic analysis for the Global Burden of Disease Study 2017. The Lancet.

[4] Folkhälsomyndigheten. Användning av tobaks- och nikotinprodukter (självrapporterat) efter ålder, kön och år. 2021.

[5] Zetterqvist M, Ramstedt M. Tobaksvanor i Sverige 2003–2018. Centralförbundet för alkohol- och narkotikaupplysning, CAN. 2019.

[6] Vos T, Lim SS, Abbafati C, Abbas KM, Abbasi M, Abbasifard M (2020). Global burden of 369 diseases and injuries in 204 countries and territories, 1990–2019: a systematic analysis for the Global Burden of Disease Study 2019. Lancet.

[7] Public Health Agency of Sweden (2021). Folkhälsans utveckling - Årsrapport.

[8] Free C, Knight R, Robertson S, Whittaker R, Edwards P, Zhou W (2011). Smoking cessation support delivered via mobile phone text messaging (txt2stop): a single-blind, randomised trial. Lancet.

[9] Scott-Sheldon LAJ, Lantini R, Jennings EG, Thind H, Rosen RK, Salmoirago-Blotcher E (2016). Text messaging-based interventions for smoking cessation: a systematic review and meta-analysis. JMIR mHealth uHealth.

[10] Whittaker R, McRobbie H, Bullen C, Rodgers A, Gu Y, Dobson R. Mobile phone text messaging and app‐based interventions for smoking cessation. Cochrane Database Syst Rev. 2019;10. 10.1002/14651858.CD006611.pub5. 10.1002/14651858.CD006611.pub5 PMC6804292 31638271

[11] Müssener U, Bendtsen M, Karlsson N, White IR, McCambridge J, Bendtsen P (2016). Effectiveness of short message service text-based smoking cessation intervention among university students: a randomized clinical trial. JAMA Intern Med.

[12] Müssener U, Linderoth C, Thomas K, Bendtsen M (2020). mHealth smoking cessation intervention among high school students: 3-month primary outcome findings from a randomized controlled trial. PLoS One.

[13] Bendtsen M, Bendtsen P, Müssener U (2021). Six-month outcomes from the NEXit junior trial of a text messaging smoking cessation intervention for high school students: randomized controlled trial with Bayesian analysis. JMIR Mhealth Uhealth.

[14] Bendtsen M, Thomas K, Linderoth C, Bendtsen P (2020). Effects of a text messaging smoking cessation intervention among online help seekers and primary health care visitors in sweden: protocol for a randomized controlled trial using a Bayesian group sequential design. JMIR Res Protoc.

[15] Lancet T (2010). CONSORT 2010. Lancet.

[16] Heatherton TF, Kozlowski LT, Frecker RC, Fagerstrom KO (1991). The Fagerstrom test for nicotine dependence: a revision of the Fagerstrom Tolerance Questionnaire. Addiction.

[17] West R, Hajek P, Stead L, Stapleton J (2005). Outcome criteria in smoking cessation trials: proposal for a common standard. Addiction.

[18] Benowitz NL, Iii PJ, Ahijevych K, Jarvis MJ, Hall S, LeHouezec J (2002). Biochemical verification of tobacco use and cessation. Nicotine Tob Res.

[19] Harris TR, Walters ST, Leahy MM (2008). Readiness to change among a group of heavy-drinking college students: correlates of readiness and a comparison of measures. J Am Coll Health.

[20] Bendtsen M (2018). A Gentle Introduction to the comparison between null hypothesis testing and Bayesian analysis: reanalysis of two randomized controlled trials. J Med Internet Res.

[21] Jackson D, Mason D, White IR, Sutton S (2012). An exploration of the missing data mechanism in an Internet based smoking cessation trial. BMC Med Res Methodol.

[22] Wood AM, White IR, Hotopf M (2006). Using number of failed contact attempts to adjust for non-ignorable non-response. J Royal Statistical Soc A.

[23] Bendtsen M (2022). Avoiding under- and overrecruitment in behavioral intervention trials using Bayesian sequential designs: tutorial. J Med Internet Res.

[24] Bendtsen M (2020). The P value line dance: when does the music stop?. J Med Internet Res.

[25] McCrabb S, Baker AL, Attia J, Skelton E, Twyman L, Palazzi K (2019). Internet-based programs incorporating behavior change techniques are associated with increased smoking cessation in the general population: a systematic review and meta-analysis. Ann Behav Med.

[26] Murray E, White IR, Varagunam M, Godfrey C, Khadjesari Z, McCambridge J (2013). Attrition revisited: adherence and retention in a web-based alcohol trial. J Med Internet Res.

[27] Gunnarsson KU, McCambridge J, Bendtsen M (2023). Reactions to being allocated to a waiting list control group in a digital alcohol intervention trial. Patient Educ Couns.

[28] Müssener U, Linderoth C, Bendtsen M (2019). Exploring the experiences of individuals allocated to a control setting: findings from a mobile health smoking cessation trial. JMIR Hum Factors.

[29] Hrobjartsson A, Thomsen ASS, Emanuelsson F, Tendal B, Hilden J, Boutron I (2012). Observer bias in randomised clinical trials with binary outcomes: systematic review of trials with both blinded and non-blinded outcome assessors. BMJ.

