# Supplementary material for: Effects of a text messaging smoking cessation intervention amongst online help-seekers and primary health care visitors: findings from a randomised controlled trial
Source: BMC Med. 2023 Oct 4;21:382. doi: 10.1186/s12916-023-03073-5 (PMC10552416; doi:10.1186/s12916-023-03073-5)
Supplement: Supplementary file 1 — Additional file 1. Recruitment criteria. Table S1. Evaluation of recruitment target criteria. [file 12916_2023_3073_MOESM1_ESM.docx]

# Additional File A - Recruitment criteria

Interim analyses indicated on 16/06/2022 that the recruitment target criteria (described in the main text) were sufficiently fulfilled such that recruitment could be paused. Supplementary Table 1 presents the evaluation of the criteria using the data available at that time. The effect criteria were clearly fulfilled for 8-week and 5-month prolonged abstinence, and 4-week point prevalence was close to being fulfilled as well. Harm and futility criteria were not close to being fulfilled. As data accrued, it was clear that effect criteria were fulfilled for all primary outcomes (see Table 2 in main text), and as such recruitment was not started again.

Table S1 - Evaluation of recruitment target criteria

|  | **Posterior probability** | | | |
| --- | --- | --- | --- | --- |
|  | **Effect** | | **Harm** | **Futility** |
|  | **OR > 1** | **OR > 1.3** | **OR < 1.3** | **1/1.3 < OR < 1.3** |
| **Three months post-randomisation (intervention versus control)** | | | | |
| 8-week prolonged abstinence | 99.9% | 97.8% | < 0.01% | 2.20% |
| 4-week point prevalence of abstinence | 95.5% | 66.2% | 0.1% | 33.7% |
| **Six months post-randomisation (intervention versus control)** | | | | |
| 5-month prolonged abstinence | > 99.9% | 99.5% | < 0.01% | 0.5% |
| 4-week point prevalence of abstinence | 92.5% | 60.2% | 0.6% | 39.2% |
